# Only a partial shield: local vancomycin postpones but does not prevent hip and knee prosthetic infections

**DOI:** 10.1007/s00402-026-06289-1

**Published:** 2026-06-18

**Authors:** Alexander Darup, Max Ettinger, Peter Savov, Sephan Brand, Gesine H Seeber, Ricarda Stauss

**Affiliations:** Division of Orthopaedics at Campus Pius-Hospital, Carl von Ossietzky University Oldenburg, School of Medicine and Health Sciences, Oldenburg, Germany

**Keywords:** Total knee arthroplasty, Total hip arthroplasty, Periprosthetic joint infection, Vancomycin powder

## Abstract

**Introduction:**

Periprosthetic joint infection (PJI) represents a severe complication following total hip arthroplasty (THA) and total knee arthroplasty (TKA). The intraarticular application of vancomycin powder is increasingly integrated into perioperative standards as an innovative strategy for PJI prevention. Yet the efficacy of this method remains a topic of debate. The aim of this retrospective cohort study was to evaluate whether topical vancomycin application reduces the incidence of PJI after primary total joint arthroplasty (TJA) and whether it is associated with an increased risk of wound complications.

**Materials and methods:**

In this retrospective monocentric cohort study, all primary THA and TKA procedures performed between January 1, 2022, and December 31, 2023, were analysed. From January 1, 2023, intraarticular vancomycin powder was implemented as part of the standard perioperative protocol for PJI prevention. The PJI rates, postoperative surgery-related complications, and time to infection were compared between the vancomycin and control groups.

**Results:**

A total of 1,499 patients were included in the study. Patients were divided into two groups: a vancomycin group (VG, *n* = 818) and a control group (CG, *n* = 681). No statistically significant group differences were observed for wound healing disorders (*p* = 0.775) or PJI rates (1.0% VG vs. 0.9% CG, *p* = 0.846). However, there was a statistically significant delay in the onset of infection in the VG compared to the CG (41.5 vs. 16.5 days, *p* = 0.006).

**Conclusion:**

Intraarticular application of vancomycin powder was not associated with a reduction in overall PJI rates after primary TJA but appeared to be associated with a longer time to infection. These findings may suggest that local vancomycin application delays the clinical manifestation of infection rather than preventing it, potentially complicating the diagnosis of PJI and limiting less invasive treatment options.

## Introduction

Periprosthetic joint infection (PJI) remains one of the most serious complications following primary hip and knee arthroplasty (THA, TKA). The incidence of PJI has been documented to range between 0.76 and 1.24% for primary THA, and 0.88–1.28% for primary TKA [1]. In high-risk patients, the reported incidence is 4–5% [2].

Current international guidelines for the prevention of PJI include standardized pre- and perioperative patient management, strict adherence to hygiene standards, and perioperative intravenous antibiotic prophylaxis prior to skin incision as a standard of care [3, 4]. These measures have substantially reduced PJI rates in primary total joint arthroplasty (TJA) to approximately 1% [5, 6]. Nevertheless, surgical site infections and PJI remain a clinical concern, and further preventive strategies are being explored. One potential complementary strategy is the local application of antibiotics. Vancomycin, which has a pronounced bactericidal effect against proliferating Gram-positive pathogens [7], is considered a particularly promising agent for this application.

However, the available evidence regarding its use in primary TJA remains inconsistent. While several studies have reported a reduction in PJI rates with local vancomycin application [8–11], others have found no significant benefit [12, 13] and have raised concerns regarding wound healing complications [12, 14]. Furthermore, the potential impact on antimicrobial resistance remains a subject of ongoing discussion [15].

Given the conflicting evidence on local vancomycin use in primary TJA, this large retrospective study evaluated its impact on the incidence of PJI as well as the time to infection. The analysis also examined whether topical vancomycin affects postoperative wound-healing complications. We hypothesized that the intraarticular application of vancomycin powder has the capacity to reduce the incidence of PJI in primary THA and TKA, without negatively affecting the postoperative complication rates and wound healing disorders.

## Materials and methods

### Study procedure and patients

**Fig. 1:**
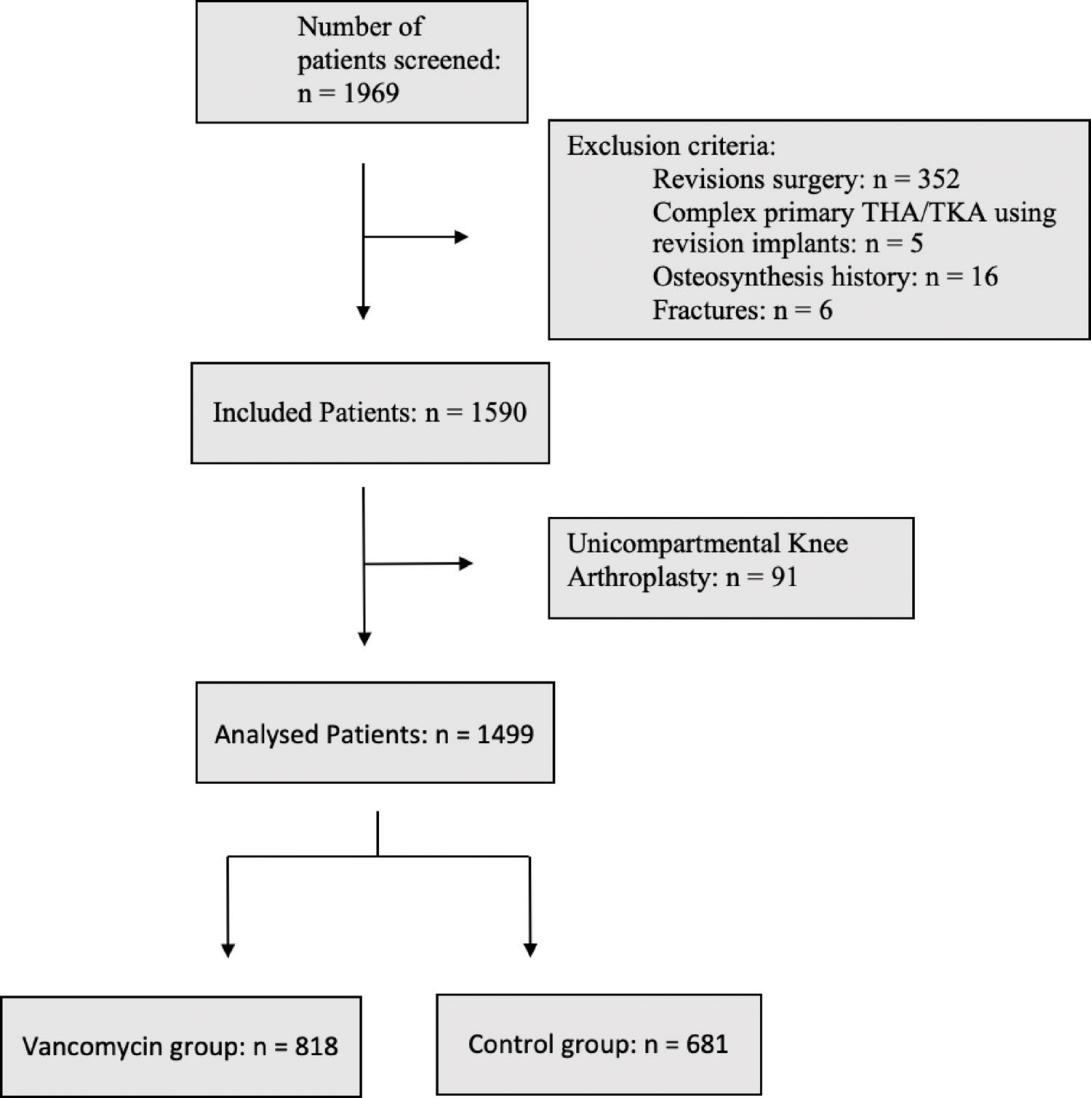
Flow chart of the study cohort

For this retrospective, monocentric cohort study, all patients who underwent primary THA or TKA between January 1, 2022, and December 31, 2023, were screened for eligibility. Following a change in the orthopaedic department’s perioperative standards for PJI prophylaxis since January 2023, 1 g of vancomycin powder was administered intraarticularly to all primary THA and TKA procedures before joint capsule closure. The study cohort was stratified into two subgroups: The vancomycin group (VG) and a control group (CG) including patients undergoing TJA before January 2023 who did not receive local antibiotics. As shown in Fig. 1, a total of 1,969 patients were screened. After excluding 470 patients based on predefined criteria — including revision procedures, complex primary surgeries, osteosynthesis history, fractures, or unicompartmental knee arthroplasty — data from 1,499 patients were included in the final analysis. Following stratification according to vancomycin administration, 818 patients were assigned to the VG and 681 patients to the CG.

### Surgical procedure

All patients received 2 g of cefazolin as the standard-of-care perioperative intravenous antibiotic prophylaxis 30 min before skin incision. Sterile skin preparation was performed according to a standardized protocol using an iodine-impregnated film (Ioban, 3 M, Saint Paul, USA). An iodine-free film was used in cases of iodine allergy. In addition, 1 g of tranexamic acid was administered intra-articularly in both groups before fascial closure.

The majority of procedures were performed by experienced surgeons who were certified as primary (*n* = 518) or senior surgeons (*n* = 892). A smaller proportion of surgeries was carried out by resident physicians (*n* = 89) for training purposes under the direct supervision of a certified surgeon. For THA procedures, an anterolateral approach according to Watson–Jones was used, and implant fixation was cementless (*n* = 896), cemented (*n* = 3), or hybrid (*n* = 23).

TKA procedures were performed using a standard medial parapatellar and cemented implant fixation using Palacos R + G (Heraeus Medical, Wehrheim, Germany). In all TKAs, a tourniquet was applied to the patient’s thigh and inflated prior to cement administration. The tourniquet was released at the end of the procedure.

### Evaluation criteria

Demographic, clinical, and intraoperative data were retrieved from the digital medical records, including age, sex, body mass index (BMI), American Society of Anesthesiologists (ASA) score, surgical procedure, surgery time, experience of the surgeon and relevant comorbidities. Postoperative complications were diagnosed by the surgeon during follow-up examinations in our outpatient clinic or emergency department.

Persistent wound secretions or wound healing disorders were assessed according to the guidelines of the Infectious Diseases Society of America (IDSA) [7, 23]. Any PJI was determined based on the criteria of the Musculoskeletal Infection Society (MSIS) [16]. The observation period for early infections was set at three months after surgery in both groups [17]. This period was chosen because longer follow-up periods can lead to higher infection rates due to bacteraemia unrelated to the operation [7, 21, 22]. The antibiotic resistance profiles of the respective patients were assessed for vancomycin resistance of the pathogens. When the indication for revision surgery was established, the surgical procedure comprised either irrigation and debridement with exchange of mobile components or a one- or two-stage prosthesis exchange. In addition, patients received intravenous antibiotic therapy tailored to the previously obtained bacteriological findings.

### Data analysis

Data analysis was performed using IBM SPSS Statistics 29 (SPSS Inc., Armonk, NY, USA). Descriptive statistics were obtained for sample characteristics at group level. Central tendency (mean) and dispersion (standard deviation [SD]) data were calculated for age and BMI. For categorical data, absolute and relative frequencies were established. The data were tested for normal distribution using the Kolmogorov-Smirnov test. Group comparisons were performed depending on the distribution type using Student´s t-test for normally distributed data or Mann-Whitney U test for non-parametric data. The Chi-square test was used to compare categorical data. Cohen’ s d was calculated as a measure of effect size for the difference in mean between groups (small effect size 0.2, medium 0.5, large 0.8) [18]. Cramer’s V was calculated as a measure of effect size for categorical variables (small effect size 0.1, medium 0.3, large 0.5) [19].

A survival analysis was performed using Kaplan–Meier estimates of cumulative survival. The Kaplan–Meier curve was chosen for its clear visualization of the time to infection, and the Log-Rank test was applied for statistical group comparison of the VG and CG. Implant survival was defined as the time from primary surgery to the diagnosis of PJI (event). Patients who were not diagnosed with PJI were censored at the end of the study period (i.e., 01 June 2024) or at the time of in-hospital death. All statistical analyses were performed with a significance level set at *p* < 0.05.

#### Ethical considerations

The study was approved by the local medical ethics committee (#2023 − 264) and conducted following the principles of the Declaration of Helsinki.

## Results

Regarding demographic characteristics, prostheses were implanted in 564 male and 935 female patients, with a mean age of 69 years. A comprehensive overview of all demographic data is provided in Table 1. Patients in both groups exhibited comparable baseline characteristics and comorbidities.

**Table 1:** Baseline characteristics of the study cohort

	Vancomycin *n* = 818	Control *n* = 681	*p*-value	Cl 95%	ES
Sex, *n* (%)			0.323	*n*/a	0.03^b^
Female	501 (61.2)	434 (63.7)			
Male	317 (38.8)	247 (36.3)			
Age, mean (SD)	69.11 (10.74)	69.82 (10.45)	0.198	– 0.37 to 1.80	0.07^a^
BMI, mean (SD)	30.14 (6.21)	30.01 (6.14)	0.696	– 0.75 to 0.50	0.02^a^
ASA score, n (%)			0.074	n/a	0.07^b^
1	18 (2.2)	15 (2.2)			
2	464 (56.7)	357 (52.4)			
3	329 (40.2)	308 (45.2)			
4	7 (0.9)	1 (0.1)			
Surgical procedure, n (%)			< 0.001*	n/a	0.13^b^
THA	457 (55.9)	465 (68.3)			
TKA	361 (44.1)	216 (31.7)			
Surgery time (min), mean (SD)	68.08 (21.98)	69.95 (23.69)	0.113	– 0.44 to 4.20	0.08^a^

*n* number of patients, *CI* Confidence interval, *ES* Effect Size Cohen’s d^a^ or Cramers-V^b^, *n/a* not applicable, *SD* Standard deviation, *BMI* body mass index, *ASA* American society of anesthesiologists, *THA* Total Hip Arthroplasty, *TKA* total knee arthroplasty, *min* minutes

* indicates statistical significance

Surgery-related complication rates did not reveal statistically significant group differences (Table 2). Delayed wound healing occurred in 14 cases (1.7%) in the VG and 13 cases (1.9%) in the CG. The PJI rates did not differ significantly between both groups (1.0% VG vs. 0.9% CG, *p* = 0.846). However, there was a statistically significant delay in the onset of infection in the VG compared to the CG (41.5 vs.16.5 days, *p* = 0.006). Vancomycin resistance did not differ significantly between both groups (*p* = 0.124).

**Table 2:** Postoperative surgery-related complication rates: wound complications, infection, and time to infection

	Vancomycin *n* = 818	Control *n* = 681	*p*-value	Cl 95%	ES
Wound complication, *n* (%)	14 (1.7)	13 (1.9)	0.775	*n*/a	0.01^b^
Infection, n (%)	8 (1.0)	6 (0.9)	0.846	n/a	0.01^b^
Time to infection (days), median (IQR)	41.50 (37.50–42.75)	16.50 (10.50–32.50)	0.006*	8.0–31.9	2.50^a^

*n* number of patients, *CI* Confidence interval, *ES* effect size Cohen’s d^a^ or Cramers-V^b^, *n/a* not applicable, *IQR* Inter Quartile Range

*indicates statistical significance

**Fig. 2:**
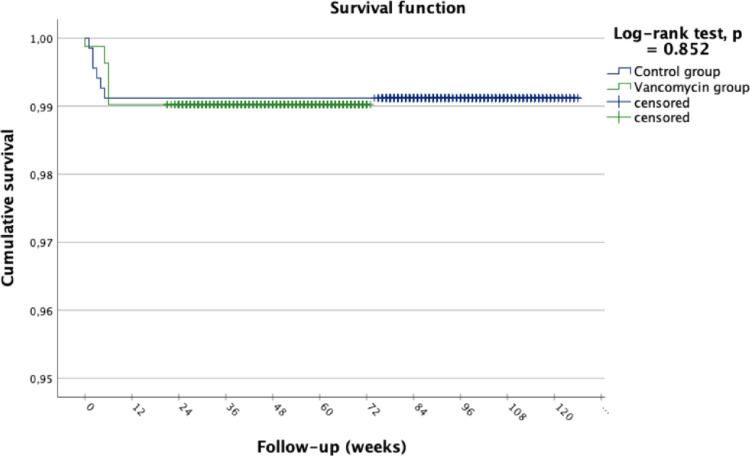
Kaplan–Meier curve showing time to infection in patients with and without vancomycin administration. There was no statistically significant difference between the groups in the Log-rank test (*p* = 0.852)

The Kaplan-Meier curve (Fig. 2) illustrates cumulative survival probability over a follow-up.

period of up to 120 weeks for the CG and VG. Both groups display highly similar survival curves, with no statistically significant differences observed (log-rank test, *p* = 0.852). All documented infections in both study groups occurred within the first six postoperative weeks, whereas no further infections were observed during the subsequent observation period.

In the subgroup diagnosed with acute PJI, the mean BMI of 34.9 kg/m^2^ was significantly higher compared to those who did not suffer a deep infection (*p* = 0.049). No statistically significant differences were found in terms of other factors including ASA score, pre-existing comorbidities, type and duration of surgery as well as surgeon experience (Table 3).

**Table 3:** Comparisons between infection and no infection in terms of age, BMI, ASA, comorbidities (diabetes mellitus II, rheumatoid arthritis), nicotine abuse, surgeon experience, surgery type and operating time

	Infection *n* = 14	No infection *n* = 1485	*p* value	Cl 95%	ES
Age, mean (SD)	67.43 (SD 9.42)	69.45 (SD 10.62)	0.478	– 3.60 to 7.61	0.20^a^
BMI, mean (SD)	34.94 (SD 3.87)	30.03 (SD 6.17)	0.003*	– 8.14 to – 1.67	0.80^a^
BMI groups, n (%)			0.049*	n/a	0.09^b^
BMI < 25	0 (0)	299 (20.1)			
BMI ≥ 25	2 (14.3)	538 (36.2)			
BMI ≥ 30	6 (42.9)	360 (24.2)			
BMI ≥ 35	4 (28.6)	181 (12.2)			
BMI ≥ 40	2 (14.3)	95 (6.4)			
BMI ≥ 50	0 (0)	12 (0.81)			
ASA score, n (%)			0.636	n/a	0.03^b^
ASA I	1 (7.1)	32 (2.2)			
ASA II	7 (50)	814 (54.8)			
ASA III	6 (42.9)	631 (42.5)			
ASA IV	0 (0)	8 (0.5)			
Comorbidities, n (%)					
Diabetes mellitus	1 (7.1)	149 (10.0)	0.720	n/a	0.01^b^
Rheumatoid arthritis	0 (0)	52 (3.5)	0.476	n/a	0.02^b^
Nicotine abuse	0 (0)	53 (3.6)	0.472	n/a	0.02^b^
Experience of the surgeon			0.516	n/a	0.03^b^
Senior chief surgeon	10 (71.4)	882 (59.4)			
Principal surgeon	4 (28.6)	514 (34.6)			
Resident	0 (0)	89 (6)			
Surgery type			0.443	n/a	0.02^b^
THA	10 (71.4)	912 (61.4)			
TKA	4 (28.6)	573 (38.6)			
Surgery time (min), mean (SD)	70.00 (SD 15.23)	68.92 (SD 22.85)	0.860	– 13.10 to 10.92	0.05^a^

*n* number of patients, *CI* confidence interval, *E* effect size Cohen’s d^a^ or Cramers-V^b^, *SD* standard deviation, *BMI* body mass index, *n/a* not applicable, *ASA* American society of anesthesiologists, *THA* total hip arthroplasty, *TKA* total knee arthroplasty, *min* minutes

*indicates statistical significance

## Discussion

The most important findings of this study are that PJI rates did not differ significantly between both investigated groups; however, a substantial delay of the onset of PJI by 25 days was observed in the VG.

The use of local antibiotic carriers in orthopaedic surgery dates back to the 1970s [20]. Recently, local application of intrawound vancomycin powder demonstrated positive effects with reduced rates of surgical site infections in spinal surgery [21] and traumatology [22]. In the current literature, results on the effect of intraarticular vancomycin application as an adjunct strategy for PJI prevention in primary TJA are inconsistent. In the present study, PJI rates were 1.0% in the VG and 0.9% in the CG, which is in line with the infection rates reported in the literature for primary TJA [23]. However, intraarticular application of vancomycin powder did not reduce PJI rates in primary THA and TKA procedures. These results are in line with findings of previous studies in the field of primary TJA [12, 13, 24, 25]. A recent study conducted by Laudet et al. analysed 1,900 primary THA and TKA procedures and reported comparable PJI rates to our study with no difference between VG and CG [13].

In contrast to that, other studies demonstrated a significant reduction of PJI rates following local application of vancomycin [7–11, 26, 27], and published meta-analyses also suggest a potential benefit [10, 28–30]. A recent meta-analysis including nine studies with 4,512 patients (2,354 in the VG and 2,158 in the CG) revealed a decrease of PJI rates in the VG by 74% in the THA and 66% in the TKA cohort, respectively [10]. Of note, comparability of the published studies is limited by discrepancies of the previously published study protocols, including different application sites, timings of applications, and vancomycin dosages [10, 12, 26]. In addition, in some studies, vancomycin was combined with povidone-iodine solution, which may result in synergistic effects [31–33]. Indeed, some studies suggest a benefit of povidone-iodine in high-risk patients and during revision surgeries [32, 33].

Interestingly, the onset of PJI was significantly delayed in the VG. This finding may reflect a temporary antimicrobial effect. Johnson et al. analysed the serum and wound vancomycin levels following local application in primary TJA. They found highly therapeutic intrawound concentrations which remained above the bactericidal threshold of 2 µg/ml for more than 60 h [34]. However, local antibiotic concentrations are known to decline rapidly over time. This may result in subinhibitory levels that transiently suppress bacterial growth without achieving complete eradication [35]. Given the observed delay in PJI onset in our study, these data suggest that local vancomycin powder application may delay acute PJI onset in primary TJA, rather than completely prevent it. Several potential mechanisms may explain this phenomenon. First, decreasing local vancomycin concentrations over time may lead to transient suppression of bacterial proliferation, followed by regrowth once concentrations fall below the minimum inhibitory concentration [15, 34]. Second, exposure to high local antibiotic concentrations may promote bacterial tolerance and persistence. This may be particularly relevant during early biofilm formation. Experimental and clinical data indicate that antibiotic selective pressure may promote biofilm-associated tolerance mechanisms [36]. This effect may be more pronounced with agents with limited biofilm penetration, such as vancomycin. Such conditions may also favour the emergence of small-colony variants. These are associated with more chronic and delayed infection courses as well as reduced antibiotic susceptibility [36, 37]. Despite the statistical significance of this finding, its clinical relevance remains uncertain. In individual cases, a delayed PJI-onset may be beneficial for the patient. Especially in multimorbid patients, a postoperative stabilization of the general condition may result in a more favourable starting point for subsequent revision procedures. However, a delayed onset of infection and potentially subclinical presentation may also complicate clinical management. A later onset of infection may obscure early clinical warning signs and delay diagnosis, thereby postponing the initiation of targeted therapy. In addition, a prolonged subclinical infection phase with progressive biofilm maturation may negatively affect the success of debridement, antibiotics, and implant retention (DAIR) procedures. These procedures show the best outcomes in early, acute infections [17]. Consequently, delayed presentation may be associated with lower DAIR success rates [38]. It may also lead to an increased need for two-stage revision procedures, and ultimately a higher likelihood of implant removal.

The systemic use of vancomycin is associated with adverse drug reactions (ADR), including nephrotoxicity, hypotension, ototoxicity, and allergic reactions [9, 34, 39]. A prospective analysis examined serum vancomycin levels following intraarticular application of vancomycin powder in primary TJA [39]. Serum concentrations remained below the therapeutic range at both 24 and 48 h postoperatively, consistent with previously published data [34]. These findings indicate that higher local concentrations with a bactericidal effect can be achieved without inducing systemic toxicity.

However, the local application of vancomycin powder may be associated with an increased risk of impaired wound healing [12, 25, 26]. In this context, Dial et al. demonstrated in a retrospective cohort study of primary THA that intrawound application of vancomycin powder was associated with a significantly increased rate of sterile wound complications [26]. In contrast, other large cohort reported no statistically significant difference in wound complication rates between patients treated with and without local vancomycin powder application [7, 13, 29]. The conflicting findings reported in the literature may be explained by several influencing factors. These include demographic characteristics (e.g., comorbidities, body mass index, and diabetes status), differences in surgical techniques, dosing and distribution of the powder preparation, and variations in perioperative wound management strategies. In the present study, postoperative wound infection rates did not differ significantly between both groups. This finding highlights the importance of a differentiated evaluation of local vancomycin application, taking into account patient- and procedure-specific factors [25].

Exposure to antibiotics is an established risk factor for the development of antimicrobial resistance. Topical application of antibiotics, such as vancomycin, is associated with a theoretical risk of resistance development; however, it is currently difficult to quantify the actual extent of this risk [15]. In vitro, vancomycin-intermediate-resistant *Staphylococcus aureus* strains have emerged under continuous exposure, but only at concentrations well above those achieved with local wound application [15, 40]. Clinical studies in spinal surgery have not reported the emergence of resistant organisms [15, 41]. In our cohort, no statistically significant difference in vancomycin resistance was observed, which may be attributable to the small sample size. All evaluated antibiograms demonstrated sensitivity. Currently, there are no reliable data on the development of resistance associated with locally applied vancomycin in TJA. This highlights the importance of using local antibiotics cautiously and selectively. Future prospective studies are warranted to investigate resistance development in the context of local antibiotics to enable evidence-based recommendations.

In our study cohort, the subgroup diagnosed with acute PJI had a significantly higher BMI compared to those without infection. This result is consistent with the current literature [42, 43]. A recent meta-analysis of over 3.2 million patient data sets showed that obesity (BMI ≥ 30 kg/m²) is associated with a 1.5-fold increased risk of PJI, and morbid obesity (BMI ≥ 40 kg/m²) is associated with a 3.3-fold increased risk of PJI [42]. The underlying reasons include increased tissue tension in the surgical area [44], restricted blood flow to the subcutaneous adipose tissue, and increased wound secretion [45]. These findings highlight the importance of optimizing patients’ modifiable risk factors prior to surgery. This is particularly relevant for those with a BMI ≥ 40 kg/m², to minimize postoperative complications. Beyond an elevated BMI, preoperative malnutrition, even among obese patients, is associated with increased rates of wound complications and PJI [46]. This finding lends support to the utilisation of screening parameters such as serum albumin [47]. Similarly, inadequate glycaemic control has been identified as an independent risk factor for PJI. This includes elevated HbA1c levels and perioperative hyperglycaemia [48]. It is hypothesised that a personalised preoperative approach and the optimisation of these factors may lead to a further reduction in the incidence of postoperative infections.

The results of this study must be interpreted with consideration for their methodological strengths and limitations. First, the retrospective, observational study design introduces potential temporal bias, as the two cohorts were collected during different time periods. To address this issue, the two groups were sequential cohorts (pre- and post-vancomycin protocol) with closely adjacent operative periods (i.e., 2022 vs. 2023) to minimize confounding due to secular trends, such as changes in pathogen epidemiology or surgical techniques.

Second, no systematic follow-up was conducted. In cases of complications or dissatisfaction, patients presented to the outpatient clinic or the emergency department. It is plausible that some patients underwent their follow-up examination at another facility due to dissatisfaction. However, this limitation is partially offset by the fact that the study was conducted at a tertiary referral centre and most surgery-related complications are referred to the primary institution.

Third, this study represents an exploratory analysis, and no a priori sample size calculation was performed. In addition, the overall incidence of early periprosthetic joint infection was low, resulting in a limited number of events, which may have reduced the statistical power to detect small to moderate differences between groups. This limitation particularly affects subgroup and survival analyses.

## Conclusion

In this cohort, the intraarticular application of vancomycin powder was not associated with a reduction in PJI rates compared to the CG. However, the time to infection was longer in the VG. Although this observation should be interpreted cautiously given the limited number of infection events, the findings of this study suggest that local application of vancomycin powder may be associated with a delayed onset of acute PJI in primary TJA. This study contributes additional observational data to the ongoing discussion on the use of local antibiotics in primary arthroplasty. However, adequately powered prospective randomized controlled trials are required to further evaluate the effect on PJI rates and the overall safety of topical vancomycin use in primary TJA.

## Data Availability

The datasets generated and analysed in this study are available from the corresponding author upon reasonable request.

## Abbreviations

ADR: Adverse drug reactions
ASA: American society of anesthesiologists
BMI: Body mass index
CG: Control group
DAIR: Debridement, antibiotics, and implant retention
IDSA: Infectious diseases society of America
MSIS: Musculoskeletal infection society
PJI: Periprosthetic joint infection
THA: Total hip arthroplasty
TJA: Total joint arthroplasty
TKA: Total knee arthroplasty
VG: Vancomycin group
